# The Specification for the Newcastle Temporary Wards

**Published:** 1914-10-31

**Authors:** 


### THE SPECIFICATION FOR THE NEWCASTLE TEMPORARY WARDS.

(By F. D. COWIESON AND CO., Charles Street, Glasgow.)

The building to be constructed with good and sound timber framing, all properly mortised and tenoned together; this to facilitate removal and re-erection at any future date; the walls and roof to be covered with best 24-gauge galvanised and corrugated iron, including all necessary ridgings and flashings, leaving the building wind and water tight at completion; a layer of insulating sheathing-felt to be fixed over the whole of the building, this to prevent draughts and variation of temperature; the whole of the building internally, including both sides of all partition walls and ceilings, to be lined with well-seasoned white pine match boarding, all to be closely laid and securely nailed, finished with the necessary mouldings at all junctions, including the necessary mouldings round door and window openings; the building to be floored throughout with 1-in. grooved and tongued white pine flooring, all to be securely laid and double nailed to the necessary joists at 18-in. centres; the building to be amply lit throughout by large-sized windows, all made to open for ventilation purposes, lower sashes being made as French casements and the upper sashes as opening fan-lights, all fitted with the necessary opening gear and other fittings; external doors, framed, lined and chamfered; internal doors, four panelled, double moulded, to include all necessary hinges and good locks with keys complete; all windows to be glazed with obscure or clear glass, as may be approved, all to be well puttied and left watertight; to include all necessary barge boards to gables as shown on drawing, also half timbers and turned finials; to include necessary gutters to eaves with downpipes to foundation level; the whole of the ceilings internally, and gables to eaves' level, to be painted two coats of best white sanitary fire-resisting water-colour paint; the remainder of the walls, doors, and windows to be sized, stained, and varnished with one coat of hard drying oak varnish; no staining or painting allowed for on floors; all exposed woodwork externally, including doors, windows, barge and fascia boards, also gutters, to be painted two coats of good oil-colour paint, picked out in two colours; the building to measure as per sizes shown on drawing, and to be 10 feet high to eaves; the ceilings to be coved to a height of at least 12 feet from ceiling to floor; all materials to be delivered free to site, site being reckoned within one mile from nearest goods station; terms, nett cash on completion.

NOTES.—With regard to the furnishing we have gone to no avoidable expense. Our lockers are simple pedestal lockers with one shelf, and are made of yellow pine, at a cost of 16s. 4d. each. The mattresses are fibre mattresses, and the sheets are cotton, not linen. It must be remembered that our site was a convenient one, and required little levelling. It also should be remembered that we have had to make no provision for sanitary accommodation.

